# The Emerging Epidemic of Obesity, Diabetes, and the Metabolic Syndrome in China

**DOI:** 10.1155/2012/178675

**Published:** 2011-09-22

**Authors:** Jia Shen, Abhinav Goyal, Laurence Sperling

**Affiliations:** ^1^Division of Cardiology, Emory University School of Medicine, Atlanta, GA 30322, USA; ^2^Department of Epidemiology and Global Health, Rollins School of Public Health, Emory University, Atlanta, GA 30322, USA; ^3^Center for Heart Disease Prevention, Emory University School of Medicine, 1365 Clifton Road, NE Building A, Suite 2200, Atlanta, GA 30322, USA

## Abstract

China is one of the fastest developing countries in the world. Rapid economic progress has resulted in changes to both diet and physical activity. New found prosperity, increased urban migration, and the adoption of sedentary lifestyles by an aging Chinese population have resulted in a dramatic shift in disease burden—from infectious to chronic. Modern Chinese find themselves increasingly afflicted with the same noncommunicable chronic diseases typical of industrialized nations. Today, cardiovascular disease is the number one cause of both morbidity and mortality, affecting both rural and urban Chinese. The rising incidence of cardiovascular disease has been fueled by an epidemic of cardiometabolic risk factors. While hypertension and smoking have received considerable spotlight, little attention has been given to obesity, diabetes, and metabolic syndrome. Their increasing prevalence is the focus of this paper.

## 1. Introduction

China is one of the fastest developing countries in the world. Since embracing market reforms in 1979, the country has experienced unequaled economic growth and has sustained an average of GDP growth of over eight percent for nearly three decades. Meanwhile, increased global trade and advances in agriculture have led to food surpluses in a country previously plagued by severe cyclical famine. This, coupled with improvements in sanitation and immunization, has dramatically raised average life expectancy from 46.6 years in 1960 to 73.1 years in 2009 [1]. Increased longevity, urbanization, and changes in traditional diets have resulted in an epidemic of cardiovascular disease (CVD). Today, CVD is the leading cause of both morbidity and mortality in China, responsible for one-third of all annual deaths [1, 2]. The increased burden of CVD can be attributed, in part, to the rapid rise in risk factors hypertension, smoking, obesity, diabetes mellitus, and metabolic syndrome. Considerable attention has already been given to both smoking and hypertension. However, while the prevalence of smoking remains high, its incidence peaked in the mid 1990s, and currently it is expected to decline over the next three decades [3]. In the meantime, rates of obesity, diabetes, and metabolic syndrome are projected to rise considerably. These cardiometabolic risk factors will play an increasingly important role in the rising CVD epidemic in China and deserve special attention.

## 2. Obesity

Obesity is an excess of body fat, defined in western populations by Body Mass Index >30 kg/m^2^ [14]. It is a known risk factor for the development of atherosclerotic cardiovascular disease, type 2 diabetes, dyslipidemia, and hypertension [15]. There is a growing body of evidence that demonstrate for any given BMI Asians have a greater percentage of body fat and a higher cardiovascular risk [10, 16–18]. In addition, Chinese people are more likely to develop central obesity, which has been associated with a higher risk of developing cardiovascular disease [19]. Based on these studies, the Working Group on Obesity in China recommended a BMI cutoff of 24 kg/m^2^ and 28 kg/m^2^, for overweight and obesity, respectively [20]. The Chinese Ministry of Health has incorporated these cutoffs in guidelines to prevent and control overweight and obesity in Chinese adults [20].

Rapid economic development and industrialization have led to changes in traditional diets and increasingly sedentary lifestyles [21, 22]. China, once considered one of the leanest populations in the world, has experienced rapidly escalating rates of overweight and obesity. A recent meta-analysis of nationally representative data by Wang [5] estimated that the prevalence of overweight and obesity rose 49.5 percent between 1992 and 2002, from 20.0 to 29.9 percent (using the WGOC cutoff for overweight and obesity) (Table 1). The rise has been greatest in urban high-income populations, such as Beijing. Similar rates have been found in other studies [4, 23], translating into an estimated 401 million overweight or obese Chinese.

In 2003, the total medical cost attributable to overweight and obesity was estimated at 2.6 billion USD, or 3.7 percent of total national medical costs [24]. The prevalence of overweight and obesity and it's economic burden on the Chinese healthcare system will only increase as greater numbers of rural Chinese move into the ranks of the urban middle class. 

## 3. Diabetes

**Figure 1:**
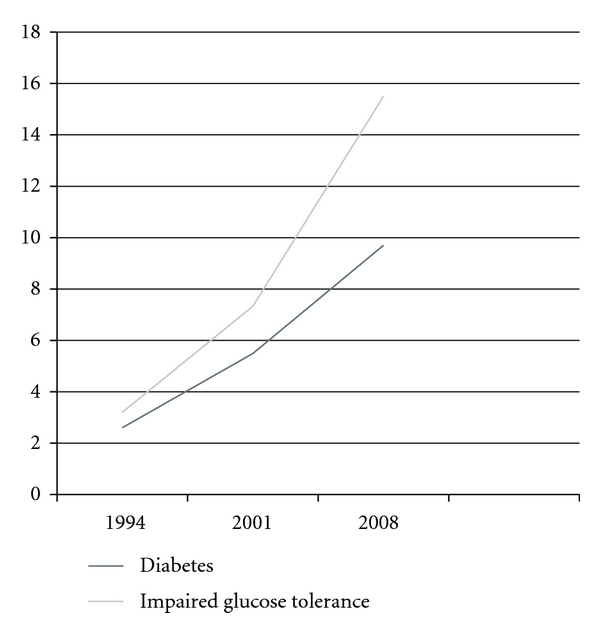
Prevalence of diabetes mellitus and impaired glucose tolerance in China 1994–2008. Adapted from [25–27].

Changes in diet and lifestyle have also led to a tremendous increase in the number of Chinese with obesity-related type 2 diabetes mellitus (T2DM). Diabetes is a major risk factor for cardiovascular disease and its prevalence has increased dramatically in the past two decades. In 1994, the prevalence of DM and IGT among individuals between 25 and 64 years was estimated to be 2.6 percent and 3.2 percent, respectively [25]. By 2001, the prevalence of diabetes and IGT had increased to 5.49 percent and 7.33 percent, respectively [26]. In 2008, the prevalence of diabetes and IGT in Chinese >20 years old had risen to 9.7 percent and 15.5 percent, respectively [27], (Figure 1).

Alarmingly, the prevalence of diabetes in China has nearly quadrupled over the last 15 years. Although the prevalence of DM and IGT remain lower than that of other industrialized nations, such as the USA, China's enormous population, estimated to be over 1.3 billion, makes it home to the largest diabetic population in the world. Today, there are 92.4 million Chinese suffering from diabetes, with an additional 148.2 million living with impaired glucose tolerance [27].

Despite the increasing prevalence of diabetes in China, rates of awareness, treatment, and control remain low. In 2000, a nationally representative study of 15,236 Chinese adults found that nearly 75 percent of diabetics were unaware of their diagnosis [6]. Of those that were aware, only 20 percent were taking prescribed medications or pursuing nonpharmacological interventions (exercise or dietary modification), of these, only 8.3 percent managed to achieve glycemic control [6] (Table 2). Similar rates of awareness were reported by Yang et al., 2010 [27]. Based on these estimates, there are currently 84.7 million Chinese with *uncontrolled* diabetes mellitus. They are at increased risk for developing long-term complications including cardiovascular disease, renal disease, peripheral neuropathy, retinopathy, and blindness. In 2007, the direct medical costs of obesity-related DM and its complication were estimated to be 26 billion USD, representing 16 percent of all Chinese medical expenditures. If current trends continue, this cost is projected to increase to 47.2 billion USD by 2030 [28].

## 4. Metabolic Syndrome

The metabolic syndrome (MetS) is a combination of interconnected risk factors for obesity, insulin resistance and glucose intolerance, dyslipidemia, and hypertension. The adult treatment panel III of the National Cholesterol Education Program (NCEP) defined a group of five clinical criteria for effective classification of MetS (Table 3). 

An individual that meets three or more of these criteria can be given the clinical diagnosis of MetS. The syndrome is associated with the development of diabetes, cardiovascular, and kidney disease, and an increased risk for mortality [29, 30]. Based on the NCEP classification, a rapidly growing epidemic of metabolic syndrome is taking place in China. In 1992, the prevalence of metabolic syndrome in China was 13.3 percent, 12.7 percent in males and 14.2 percent in females [31]. By 2000, the prevalence of metabolic syndrome had increased to 15.1 percent, 13.6 percent in men and 16.6 percent in women [32]. This prevalence rate was determined using the modified Asian criteria for waist circumference, based on studies which have shown that Asians suffer from the same risk of cardiovascular disease development at smaller waist circumferences, 90 cm for men and 80 cm for women [33, 34]. In 2000, 64 million Chinese had metabolic syndrome, this number increases to 71 million using the modified ATP III criteria for Asian populations [35]. 

**Figure 2:**
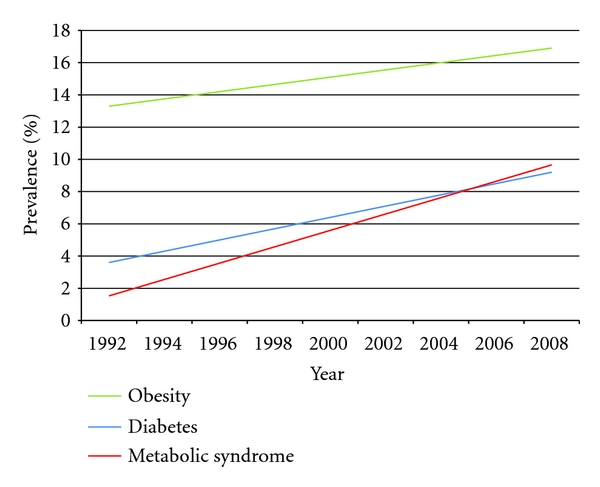
Prevalence of obesity, diabetes and, metabolic syndrome trends over time 1992–2008.

Numerous studies have shown that the prevalence of metabolic syndrome increases with age [35–37]. This finding has important implications for China, whose enormous population is aging rapidly as an unintended consequence of the “one child policy” adopted by the government in 1978. The population of Chinese seniors grows by 3.3 percent annually. By 2050, 30 percent of the population will be over the age of 65 years [38]. The increasing prevalence of MetS among elderly Chinese was demonstrated in a Hong Kong-based population study by Thomas [39]. Of participants aged 25–29 years, only 3.1 percent had MetS, compared to 41.0 percent of those aged over 70 years. The age- and gender-adjusted prevalence was 21.2 percent. This approaches prevalence rates found in other industrialized nations [40, 41]. The high prevalence of MetS among Hong Kong Chinese forewarns a rapidly increasing problem in Mainland, China. Hong Kong, with its vibrant economy fostered by decades of British occupation has already gone through the rapid socioeconomic changes the rest of the country is currently experiencing. In addition, the rapid influx of rural Chinese into the country's overcrowded megacities will only accelerate the rapid rise in MetS and related complications [42], (Figure 2).

## 5. Conclusion

China is a middle-income country in rapid economic and epidemiologic transition. Decades of economic development have led to dramatic changes in life expectancy, lifestyle, and diet. An older more sedentary population finds itself increasingly more burdened by diseases of its newfound wealth obesity, diabetes, and metabolic syndrome, which already approach those found in industrialized nation in both Europe and the USA. The increasing prevalence of cardiometabolic risk factors has resulted in an unprecedented rise in the incidence of cardiovascular disease. The incidence of CVD is predicted to rise by at least 50% in the next 20 years, with an additional 23 percent increase resulting from trends in cardiometabolic risk factors [43]. Perhaps even more alarming, Chinese are less likely to be diagnosed and less likely to gain control of their chronic disease [6, 44] and have limited access to quality healthcare services [45, 46]. Furthermore, the Chinese healthcare system, with its historical focus on treating infectious diseases, is ill equipped to handle the rising epidemic of cardiovascular disease [47]. The Chinese have proven themselves capable of rapid large-scale socioeconomic change. However, the ability of the government to respond and control such an epidemic will depend on whether or not it approaches the task with the same vigor it has approached economic development. While China can benefit from the rich body of existing research on cardiometabolic disease, more studies on Chinese-specific risk factors are needed.

## Figures and Tables

**Table 1:** Obesity trends 1992–2002 [4].

Classification BMI Kg/m^2^	Prevalence (%)		Percent change 1992–2002	Estimated number of overweight & obese chinese*
	1992	2002		
Overweight 24.0–27.9	16.4	22.8	39.0%	305,748,000
Obese > 28.0	3.6	7.1	97.2%	95,211,000

Adapted from [5].

*Based on a population statistics reported by the Chinese Bureau of Statistics, 2011.

**Table 2:** Awareness, treatment, and control of diabetes in Chinese^t^.

Population	Awareness	Treatment	Control
China	23.7%	4.81%	0.40%

^t^Percentage of total diabetics.

Adapted from [6].

**Table 3:** NCEP ATP III diagnostic criteria for metabolic syndrome [7–9].

Criteria	Category
Elevated fasting glucose	≥110 mg/dL or use of antidiabetic medications±
Elevated triglycerides	≥150 mg/dL
Reduced HDLC	<40 mg/dL in men<50 mg/dL in women
Elevated blood pressure	≥130 mm Hg or ≥85 mm Hg DBP or use of antihypertensive medication
Elevated waist circumference	>102 cm in men>88 cm in women

WHO modified Asian waist circumference criteria [10]	

Elevated waist circumference	>90 cm in men>80 cm in women

±WHO classifies fasting blood glucose >100 mg/dL as elevated.

Adapted from [11–13].

## References

[1] The World Bank (2011). *Development Indicators 2011*.

[2] Leeder S, Raymond S, Greenberg H (2004). *A Race Against Time: The Challenge of Cardiovascular Disease in Developing Countries*.

[3] Ministry of Health, People’s Republic of China (2006). China’s Smoking & Health Report.

[4] Tian H, Xie H, Song G, Zhang H, Hu G (2009). Prevalence of overweight and obesity among 2.6million rural Chinese adults. *Preventive Medicine*.

[5] Wang Y, Mi J, Shan XY, Wang QJ, Ge KY (2006). Is China facing an obesity epidemic and the consequences? The trends in obesity and chronic disease in China. *International Journal of Obesity*.

[6] Hu D, Fu P, Xie J (2008). Increasing prevalence and low awareness, treatment and control of diabetes mellitus among Chinese adults: the InterASIA study. *Diabetes Research and Clinical Practice*.

[7] World Health Organization Department of Non-Communicable Disease Surveillance (1999). Definition, diagnosis and classification of diabetes mellitus and its complications.

[8] World Health Organization (2000). Preventing and managing the Global Epidemic—report of a WHO Consultation. *WHO Technical Report Series*.

[9] Cleeman JI (2001). Executive summary of the third report of the national cholesterol education program (NCEP) expert panel on detection, evaluation, and treatment of high blood cholesterol in adults (adult treatment panel III). *Journal of the American Medical Association*.

[10] Barba C, Cavalli-Sforza T, Cutter J (2004). Appropriate body-mass index for Asian populations and its implications for policy and intervention strategies. *Lancet*.

[14] The World Health Organization (1995). Physical status: the use and interpretation of anthropometry—report of a WHO expert committee. *WHO Technical Report*.

[15] Grundy SM, Becker D, Clark LT (2002). Detection, evaluation, and treatment of high blood cholesterol in adults (Adult Treatment Panel III) final report. *Circulation*.

[16] Zhou BF (2002). Predictive values of body mass index and waist circumference for risk factors of certain related diseases in Chinese adults–study on optimal cut-off points of body mass index and waist circumference in Chinese adults. *Biomedical and Environmental Sciences*.

[17] Misra A (2003). Revisions of cutoffs of body mass index to define overweight and obesity are needed for the Asian-ethnic groups. *International Journal of Obesity*.

[18] Inoue S, Zimmet P, Caterson I (2000). *The Asia-Pacific Perspective: Redefining Obesity and its Treatment*.

[19] Thomas GN, Ho SY, Lam KSL, Janus ED, Hedley AJ, Tai HL (2004). Impact of obesity and body fat distribution on cardiovascular risk factors in Hong Kong Chinese. *Obesity Research*.

[20] Chen C, Lu FC (2006). The guidelines for prevention and control of overweight and obesity in Chinese adults. *Biomedical and Environmental Sciences*.

[21] Bell AC, Ge K, Popkin BM (2002). The road to obesity or the path to prevention: motorized transportation and obesity in China. *Obesity Research*.

[22] Wang H, Du S, Zhai F, Popkin BM (2007). Trends in the distribution of body mass index among Chinese adults, aged 20-45 years (1989-2000). *International Journal of Obesity*.

[23] Jia WP, Wang C, Jiang S, Pan JM (2010). Characteristics of obesity and its related disorders in china. *Biomedical and Environmental Sciences*.

[24] Zhao W, Zhai Y, Hu J (2008). Economic burden of obesity-related chronic diseases in Mainland China. *Obesity Reviews*.

[25] Pan X, Yang W, Liu J (1994). Prevalence of diabetes and its risk factors in China 1994. National Diabetes Prevention and Control Cooperative Group. *Diabetes Care *.

[26] Gu D, Reynolds K, Duan X (2003). Prevalence of diabetes and impaired fasting glucose in the Chinese adult population: international collaborative study of cardiovascular disease in asia (InterASIA). *Diabetologia*.

[27] Yang W, Lu J, Weng J (2010). Prevalence of diabetes among men and women in China. *New England Journal of Medicine*.

[28] Wang W, McGreevey WP, Fu C (2009). Type 2 diabetes mellitus in China: a preventable economic burden. *American Journal of Managed Care*.

[29] Isomaa B, Almgren P, Tuomi T (2001). Cardiovascular morbidity and mortality associated with the metabolic syndrome. *Diabetes Care*.

[30] Chen J, Muntner P, Hamm LL (2004). The Metabolic Syndrome and Chronic Kidney Disease in U.S. Adults. *Annals of Internal Medicine*.

[31] Further Study of Risk Factors for Stroke and Coronary Heart Disease Cooperation Group (2002). The prevalence of metabolic syndrome in a 11 provinces cohort in China. *Zhonghua Yu Fang Yi Xue Za Zhi*.

[32] Gu D, Gupta A, Muntner P (2005). Prevalence of cardiovascular disease risk factor clustering among the adult population of China: results from the International Collaborative Study of Cardiovascular Disease in Asia (InterAsia). *Circulation*.

[33] Tan CE, Ma S, Wai D, Chew SK, Tai ES (2004). Can we apply the national cholesterol education program adult treatment panel definition of the metabolic syndrome to absians?. *Diabetes Care*.

[34] Lee WY, Park JS, Noh SY, Rhee EJ, Kim SW, Zimmet PZ (2004). Prevalence of the metabolic syndrome among 40,698 Korean metropolitan subjects. *Diabetes Research and Clinical Practice*.

[35] Hildrum B, Mykletun A, Hole T, Midthjell K, Dahl AA (2007). Age-specific prevalence of the metabolic syndrome defined by the International Diabetes Federation and the National Cholesterol Education Program: the Norwegian HUNT 2 study. *BMC Public Health*.

[36] Kraja AT, Borecki IB, North K (2006). Longitudinal and age trends of metabolic syndrome and its risk factors: the family heart study. *Nutrition and Metabolism*.

[37] Ford ES, Giles WH, Dietz WH (2002). Prevalence of the metabolic syndrome among US adults: findings from the third national health and nutrition examination survey. *Journal of the American Medical Association*.

[38] The United Nations Population Division (2001). World Population Ageing: 1950–2050.

[39] Thomas GN, Ho SY, Janus ED, Lam KSL, Hedley AJ, Lam TH (2005). The US national cholesterol education programme adult treatment panel III (NCEP ATP III) prevalence of the metabolic syndrome in a Chinese population. *Diabetes Research and Clinical Practice*.

[40] Ervin RB (2009). Prevalence of metabolic syndrome among adults 20 years of age and over, by sex, age, race and ethnicity, and body mass index: United States. *National health statistics reports*.

[41] Grundy SM (2008). Metabolic syndrome pandemic. *Arteriosclerosis, Thrombosis, and Vascular Biology*.

[42] Ebrahim S, Kinra S, Bowen L (2010). The effect of rural-to-urban migration on obesity and diabetes in india: a cross-sectional study. *PLoS Medicine*.

[43] Moran A, Gu D, Zhao D (2010). Future cardiovascular disease in China Markov model and risk factor scenario projections from the coronary heart disease Policy Model-China. *Circulation: Cardiovascular Quality and Outcomes*.

[44] Dong GH, Sun ZQ, Zhang XZ (2008). Prevalence, awareness, treatment & control of hypertension in rural Liaoning province. *China Indian Journal of Medical Research*.

[45] Ministry of Health, People’s Republic of China (1999). Report on the 1998 national health services survey results.

[46] Anand S, Fan VY, Zhang J (2008). China’s human resources for health: quantity, quality, and distribution. *Lancet*.

[47] Cheng MH (2010). Asia-Pacific faces diabetes challenge. *Lancet*.

